# Single-cell Spatial Multiomics: Technologies, Methods, and Biological Applications

**DOI:** 10.1093/gpbjnl/qzag012

**Published:** 2026-02-09

**Authors:** Dong Xing (邢栋), Rong Fan, Fangqing Zhao (赵方庆)

**Affiliations:** Biomedical Pioneering Innovation Center (BIOPIC) and School of Life Sciences, Peking University, Beijing 100871, China; Beijing Advanced Innovation Center for Genomics (ICG), Peking University, Beijing 100871, China; Department of Biomedical Engineering, Yale University, New Haven, CT 06520, USA; Yale Stem Cell Center and Yale Cancer Center, Yale School of Medicine, New Haven, CT 06520, USA; Department of Pathology, Yale School of Medicine, New Haven, CT 06520, USA; Human and Translational Immunology, Yale School of Medicine, New Haven, CT 06520, USA; Institute of Zoology, Chinese Academy of Sciences, Beijing 100101, China; Key Laboratory of Systems Biology, Hangzhou Institute for Advanced Study, University of Chinese Academy of Sciences, Hangzhou 310024, China; University of Chinese Academy of Sciences, Beijing 100049, China

Advances in single-cell genomics have profoundly transformed our understanding of cellular heterogeneity, yet the loss of spatial context has remained a major limitation in interpreting cell states and regulatory programs within intact tissues. In recent years, spatially resolved transcriptomics and epigenomics have begun to bridge this gap by enabling the localization of molecular profiles within their native tissue architectures. Building upon these developments, single-cell spatial multiomics approaches are now emerging as a powerful paradigm, allowing multiple molecular layers, such as gene expression, chromatin accessibility, epigenetic modifications, and genome organization, to be measured in a spatially informed manner. Together, these technologies provide unprecedented opportunities to dissect how molecular regulation is orchestrated across space, cell types, and tissue microenvironments, thereby offering deeper insights into development, physiology, and disease.

The “Spatial Multiomics” Special Issue of *Genomics, Proteomics & Bioinformatics* is dedicated to recent advances in single-cell spatial multiomics, bringing together studies that span methodological innovations, computational analyses, and biological applications. The issue comprises one Research Highlight, one Review, five Original Research articles, and three Method papers, collectively reflecting the rapid evolution and growing diversity of this field.

In the Research Highlight, Xiao and Ma [1] provided a perspective on a recently published spatial epigenomics study, highlighting how joint spatial profiling of DNA methylation and transcription within the same tissue section enables direct links between epigenetic states and gene expression in their native spatial context. In the Review article, He et al. [2] summarized the evolution of spatial omics technologies and their applications to lung cancer, illustrating how spatially resolved multiomics enables systematic dissection of tumor heterogeneity, cellular interactions, and immune niches that are inaccessible to bulk or dissociated single-cell approaches, while outlining key challenges and translational opportunities.

In the first Original Research article, Chen et al. [3] constructed a comprehensive spatial transcriptomic atlas of mammalian lung development, integrating spatial gene expression with regulatory and cell–cell communication analyses to reveal how transcriptional programs, developmental trajectories, and tissue architecture are coordinately organized across key stages of pulmonary morphogenesis. Qian et al. [4] applied spatial ATAC-seq to human breast cancer tissues to map intratumoral epigenetic heterogeneity *in situ*, revealing spatially organized regulatory programs, tumor subclones, and microenvironmental niches that underlie divergent tumor behaviors and therapeutic responses. Shi et al. [5] generated a single-cell and spatially resolved multiomics atlas of human esophageal squamous cell carcinoma, integrating transcriptomic and metabolic profiling to uncover a fibroblast–tumor epithelial niche that forms an immune-excluded barrier and promotes tumor progression. Sha et al. [6] constructed a spatial transcriptomic atlas of the human decidua in early pregnancy, revealing spatially organized immune niches and regulatory programs, whose disruption underlies impaired maternal–fetal immune tolerance in recurrent pregnancy loss. Chen et al. [7] integrated single-nucleus and spatial transcriptomics to delineate the functional programs and developmental trajectories of bacteriocytes in deep-sea mussels, revealing how conserved regulatory networks coordinate immune, metabolic, and developmental processes underlying host–symbiont adaptation in extreme environments.

In addition, this special issue includes three Method papers that expand the technical and analytical toolboxes for spatial multiomics research. Liu et al. [8] introduced Histo-LCM-Hi-C, a spatially resolved Hi-C approach that combines histological labeling with laser capture microdissection to enable high-resolution mapping of three-dimensional chromatin architecture in spatially defined and rare cell populations within intact tissues. Liao et al. [9] presented HyperSTAR, a hypergraph learning-based approach that captures higher-order spatial relationships to robustly identify spatial domains across varying resolutions and spatial omics platforms, enabling improved tissue structure delineation and tumor microenvironment analysis. Zhen et al. [10] introduced DisConST, a distribution-aware contrastive learning approach that integrates spatial information, transcriptomic profiles, and cell-type proportions to robustly identify spatial domains across diverse spatial transcriptomics platforms and biological contexts.

Collectively, the contributions in this special issue highlight the rapid maturation of single-cell spatial multiomics as a field. By uniting methodological innovation with diverse biological and clinical applications, these studies underscore the power of spatially resolved, multi-layered measurements to link cellular states, tissue architecture, and functional outcomes. We anticipate that the approaches and resources presented here will further advance spatial multiomics research and accelerate its impact across basic biology and translational medicine.

## CRediT author statement

**Dong Xing:** Conceptualization, Writing – original draft, Writing – review & editing. **Rong Fan:** Writing – review & editing. **Fangqing Zhao:** Writing – review & editing. All authors have read and approved the final manuscript.
